# Layer-specific Developmental Changes in Excitation and Inhibition in Rat Primary Visual Cortex

**DOI:** 10.1523/ENEURO.0402-17.2017

**Published:** 2017-12-14

**Authors:** Roberta Tatti, Olivia K. Swanson, Melinda S. E. Lee, Arianna Maffei

**Affiliations:** 1 Department of Neurobiology and Behavior, SUNY – Stony Brook, Stony Brook, NY; 2 Graduate Program in Neuroscience, SUNY – Stony Brook, Stony Brook, NY

**Keywords:** Development, excitation, experience, inhibition, visual cortex

## Abstract

Cortical circuits are profoundly shaped by experience during postnatal development. The consequences of altered vision during the critical period for ocular dominance plasticity have been extensively studied in rodent primary visual cortex (V1). However, little is known about how eye opening, a naturally occurring event, influences the maturation of cortical microcircuits. Here we used a combination of slice electrophysiology and immunohistochemistry in rat V1 to ask whether manipulating the time of eye opening for 3 or 7 d affects cortical excitatory and inhibitory synaptic transmission onto excitatory neurons uniformly across layers or induces laminar-specific effects. We report that binocular delayed eye opening for 3 d showed similar reductions of excitatory and inhibitory synaptic transmission in layers 2/3, 4, and 5. Synaptic transmission recovered to age-matched control levels if the delay was prolonged to 7 d, suggesting that these changes were dependent on binocular delay duration. Conversely, laminar-specific and long-lasting effects were observed if eye opening was delayed unilaterally. Our data indicate that pyramidal neurons located in different cortical laminae have distinct sensitivity to altered sensory drive; our data also strongly suggest that experience plays a fundamental role in not only the maturation of synaptic transmission, but also its coordination across cortical layers.

## Significance Statement

Studies in patients born with bilateral or unilateral cataracts, delaying the onset of vision, have reported profoundly altered visual function. This effect is thought to depend on altered maturation of visual cortical circuits, although the mechanisms for these changes are currently unknown. We report significant differences in the effect of binocular and monocular delayed eye opening on excitatory and inhibitory synaptic transmission onto excitatory neurons in rat V1. Binocular delays had transient, but similar, effects across layers, whereas monocular delays showed long-lasting, laminar-specific changes. Our data suggest that differences in coordination of synaptic transmission across visual cortical layers at the time of eye opening may contribute to the distinct functional effects of binocular or monocular delays in early vision in patients.

## Introduction

Cortical layers in primary visual cortex (V1) form recurrent microcircuits activated by different sets of inputs (LeVay and Gilbert, 1976; Peters and Feldman, 1977; Martin, 2002; Wang et al., 2013). It is widely accepted that local circuits within each layer of V1 mature in an experience-dependent fashion (Fagiolini et al., 1994; Katz and Shatz, 1996). Indeed, synaptic transmission in V1 can be strongly influenced by the time of eye opening (Garkun and Maffei, 2014) and is exquisitely sensitive to changes in visual drive (Tagawa et al., 2005; Smith and Trachtenberg, 2007). As excitatory and inhibitory circuits mature (Desai et al., 2002; Morales et al., 2002; Heinen et al., 2004), their relative relationship [excitatory/inhibitory (E/I) ratio, or balance] contributes to tuning circuit excitability, a process that is crucial for the development of healthy visual function (Hensch and Fagiolini, 2005). It is currently unknown whether the E/I balance onto excitatory neurons in the distinct layers of V1 may be influenced by the time of eye opening and whether this parameter matures uniformly across layers.

Here we used brief (3-d) and long (7-d) delays in binocular and monocular eye opening to shift the onset of vision and assess the effect of these manipulations on the E/I balance of pyramidal neurons located in all layers of V1. Our analysis straddled the developmental window between eye opening [postnatal day 14 (P14)] and the peak of the critical period (P30). Immunohistochemistry and electrophysiology were used to assess the laminar distribution of two distinct populations of inhibitory neurons and determine the E/I ratio of synaptic currents onto pyramidal neurons.

We report extensive layer-specific differences in postnatal maturation of synaptic transmission and in the laminar distribution of one class of inhibitory neurons during normal postnatal development. Although the prevalence of PV^+^ and SST^+^ inhibitory neurons was not sensitive to manipulations of visual drive, synaptic transmission was significantly affected. A 3-d delay in binocular eye opening (bDEO) decreased excitatory drive in L2/3, L4, and L5 and prevented the age-dependent increase in inhibition in L2/3, 4, and 5. Interestingly, if eye opening was delayed binocularly for 7 d, synaptic transmission in all layers was comparable to that of age-matched littermates, suggesting the engagement of compensatory mechanisms. Monocular delayed eye opening (mDEO) had profoundly different effects from bDEO. A 3-d mDEO induced laminar-specific changes in excitatory and inhibitory drive onto pyramidal neurons, with significant shifts in E/I balance only in L2/3 and L4. A longer mDEO induced additional changes that further disrupted coordination across layers. Together, our data indicate that laminar circuits within neocortex show distinct patterns of maturation that are coordinated by binocular visual drive. Although symmetric delays in the onset of vision reduce synaptic drive onto pyramidal neurons without affecting the E/I balance, asymmetric delays alter the E/I balance and disrupt coordination across layers, unveiling layer-specific mechanisms of refinement. Our results indicate that the laminar difference in sensitivity to altered visual drive of local circuits in V1 should be taken into consideration when devising therapeutic approaches for the recovery of healthy visual function.

## Materials and Methods

All experimental procedures were approved by the Institutional Animal Care and Use Committee of the authors’ institution and followed the guidelines of the National Institutes of Health.

### Immunohistochemistry

**Figure 1.**
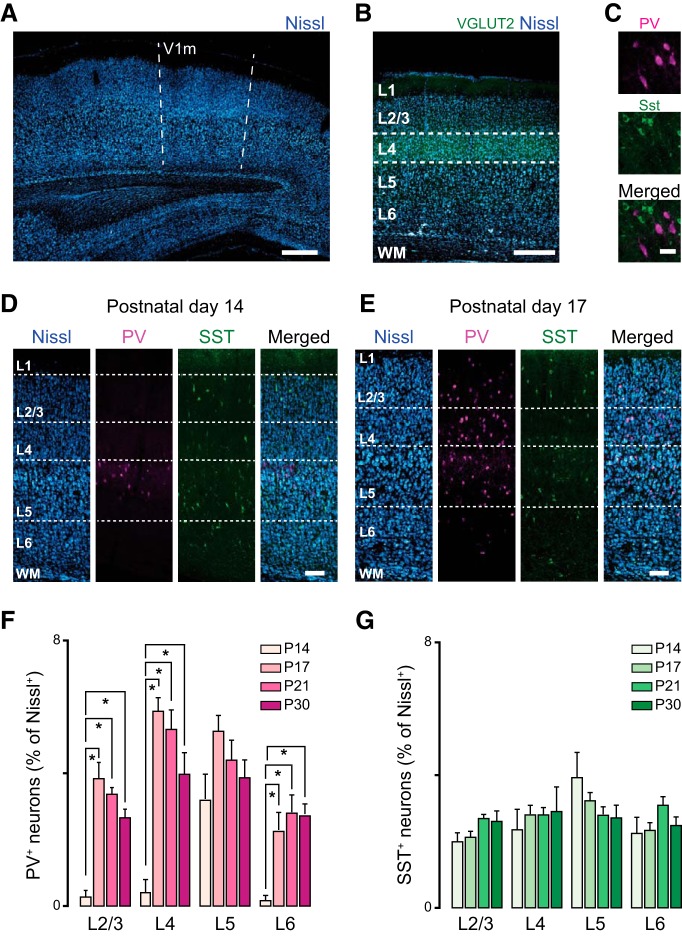
Age-dependent expression of interneurons in rat primary visual cortex. ***A***, Confocal image taken with a 01× objective showing the location of the monocular portion of rat V1 (V1m). The landmarks of the white matter were used to identify V1m. Scale bar: 500 µm. ***B***, Adjacent sections were processed with antibody staining for the vesicular glutamate transporter-2 (VGLUT2, green) and fluorescent Nissl (blue) to measure L4 distance from the pia and identify layers. Scale bar: 400 µm. ***C***, High-magnification image acquired with a 20× objective showing PV^+^ (upper panel; magenta) and SST^+^ (SST; middle panel; green) expressing neurons; the merged image is shown in the lower panel. Scale bar: 35 µm. ***D***, From left to right: confocal images showing the distribution of Nissl, PV^+^, and SST^+^ neurons at P14 across the cortical mantle of rat visual cortex. Scale bar: 125 µm. ***E***, From left to right: confocal images showing the distribution of Nissl, PV^+^, and SST^+^ neurons at P17. Scale bar: 125 µm. ***F***, Percentage of PV^+^ interneurons across V1 cortical layers quantified in four postnatal age groups (P14, P17, P21, P30). ***G***, Percentage of SST^+^ interneurons across V1 cortical layers quantified in four postnatal age groups. 10 coronal slices from 4 rats were used for quantification. Data are mean ± SEM. Statistical significance was calculated using ANOVA, and *p* values were corrected for multiple comparisons.

Rats of both sexes (Long-Evans; Charles River) were deeply anesthetized with an intraperitoneal injection of a mixture of 70 mg/kg ketamine, 3.5 mg/kg xylazine hydrochloride, and 0.7 mg/kg acepromazine maleate, then transcardially perfused with PBS, followed by 4% paraformaldehyde (PFA) in 0.1 m phosphate buffer at 4°C (PBS, pH 7.3). Brains were then dissected out and postfixed overnight in 4% PFA. During the following days, brains were cryoprotected with 30% sucrose and embedded in Tissue Tek OCT. Coronal slices (50 µm) containing V1 were prepared with a cryostat (MICROM HM505SE) and collected in PBS (0.1 m). Double immunostaining for parvalbumin (PV^+^) and somatostatin (SST^+^) and single immunostaining for the vesicular glutamate transported type 2 (VGluT2, which labels thalamocortical axon terminals) were performed in parallel on alternate slices. Slices were incubated overnight in Tris-buffered saline with Tween (TBST) solution containing the primary antibodies (mouse anti-PV: 1:2000, Swant, cat. no. 235, RRID: AB_10000343, rabbit anti-SST: 1:200, Santa Cruz Biotechnology, cat. no. sc-13099, RRID: AB_2195930), then immunoreacted with secondary antibodies (1:200): Alexa Fluor 594 goat anti-mouse (Life Technologies, cat. no. A11032, RRID: AB_141672) and Alexa Fluor 488 goat anti-rabbit (Life Technologies, cat. no. A11034, RRID; AB_2576217). To label VGluT2-expressing terminals, slices were immunostained with an anti-VGLUT2 antibody (rabbit anti-VGLUT2, 1:1000, Synaptic Systems, cat. no. 135 403, RRID: AB_887883), followed by incubation in the secondary antibody Alexa Fluor 488 goat anti-rabbit (1:200; Life Technologies, cat. no. A11034, RRID; AB_2576217). VGluT2 labeling was used to facilitate the identification of L4 within V1m (Fig. 1*A*, *B*). All slices were counterstained with fluorescent Nissl (Neurotrace 435/455, Thermo Fisher Scientific, cat. no. N21479) and mounted with Fluoromount-G (SouthernBiotech, cat. no. 0100-01).

For each animal, we analyzed 10 slices taken every 100 µm to ensure sampling of V1m at different levels along the rostro-caudal axis. Images containing the monocular region of V1m were acquired with a confocal laser-scanning microscope (Olympus Fluoview 1000) using a 10× objective. V1m was identified using the coordinates from the atlas for the rat brain (Paxinos and Watson, 2007) corrected for the age difference in the lambda-bregma distance, as well as the morphology of the white matter tract.

Projection images were made in ImageJ. We first outlined regions of interest (ROIs) containing each layer in ImageJ. The number of Nissl^+^ neurons within each layer was quantified using the plugin Image-based Tool for Counting Nuclei (ITCN). Once the diameter of a cell and the minimum distance between cells was chosen, it was maintained constant to analyze all acquired images. All images were acquired using a 10× objective and the same microscope settings, so that the measuring parameters were constant across animals. To quantify the percentage of PV^+^ or SST^+^ neurons in each layer of V1m, we used the plugin cell counter. Cell number was expressed as percentage of Nissl-PV^+^ (or SST^+^) neurons.

### Delayed eye opening

Eye opening was delayed either with binocular eyelid suture (bDEO) or monocular eyelid suture (mDEO). Both procedures were started before the naturally occurring eye opening at P13–P14 and maintained for 3 or 7 d (Maffei et al., 2004; Maffei and Turrigiano, 2008). To perform eyelid sutures, animals were anesthetized with an IP injection of a mixture containing 70 mg/kg ketamine, 3.5 mg/kg xylazine hydrochloride, and 0.7 mg/kg acepromazine maleate. The area around the eyelid was disinfected with betadine, and sutures were placed using 6-0 polyester sterile thread. At the end of the procedure, animals were allowed to recover while maintaining body temperature with a heating pad and were placed back in their home cage only when fully alert. The sutured eyes were checked twice daily under a dissection microscope to ensure full closure and lack of infection. Only animals whose sutures were perfectly preserved were used for recordings.

### Slice electrophysiology

Long-Evans rats of both sexes were anesthetized with isoflurane and decapitated. Acute coronal slices (300 µm) containing the monocular portion of V1 (V1m) were prepared as described (Maffei et al., 2004). To identify V1m, we followed the criteria used during confocal imaging sessions. Brain slices were incubated in standard artificial cerebrospinal fluid (ACSF) at room temperature for at least 1 h before recordings. ACSF contained the following (in mm): 126 NaCl, 3 KCl, 25 NaHCO_3_, 1 NaHPO_4_, 2 MgSO_4_, 2 CaCl_2_, 14 dextrose, pH 7.4, when bubbled with mixed 95% CO_2_ and 5% O_2_. For recordings, a single visual cortical slice was transferred to the recording chamber and perfused with standard ACSF. Recordings were performed at room temperature. Visually guided whole-cell recordings were obtained using an IR-DIC microscope (Olympus BX51). Recordings were performed using borosilicate glass pipettes with resistance of 3–5 MΩ and filled with an intracellular solution containing (mm): 100 Cs-sulfate, 20 KCl, 10 Hepes, 4 Mg-ATP, 0.3 Na-GTP, 10 phosphocreatine, and 0.4% biocytin. Osmolarity was adjusted to 295 mOsm with sucrose. Spontaneous EPSCs (sEPSCs) were recorded by holding the neurons at the reversal potential for GABA_A_-mediated currents (–50 mV), and spontaneous IPSCs (sIPSCs) were recorded at the reversal potential for AMPA–NMDA-mediated currents (10 mV). To assess the quality of the recordings, input resistance was monitored throughout recordings. Neurons with >20% change in input resistance were not included in the analysis. Patch-clamp recordings were obtained from pyramidal neurons in L2/3, L4, L5, and L6.

Pyramidal cell morphology and laminar location of all neurons included in this study were confirmed by *post hoc* biocytin staining. Briefly, slices were fixed for at least 48 h in PBS (pH 7.4) containing 4% PFA. Thereafter, slices were transferred into PBS and incubated overnight with streptavidin Alexa Fluor 594 (1:1000) and fluorescent Nissl counterstaining 435/455 (Thermo Fisher Scientific, cat. no. N21479). A confocal laser-scanning microscope (Olympus Fluoview 1000) was used to acquire images of recorded neurons and assess their morphology and laminar location. After the confocal imaging procedures, slices were unmounted and developed with diaminobenzidine to reconstruct their entire morphology in bright field using the software Neurolucida (MicroBrightField).

### Statistical analysis

All data are presented as average ± standard error of the mean (SEM) for the number of neurons indicated. In this study, we used parametric (*t* test and one-way ANOVA) and nonparametric tests [Mann-Whitney (MW) *U* test and Kruskal-Wallis (KW) ANOVA] after assessment of normality with the Shapiro-Wilk test. To determine differences across ages and layers, we used KW ANOVA followed by *post hoc* MW *U* test. Bonferroni correction was used to correct for multiple comparisons [critical *p* value (α)/number of comparisons where α = 0.05, and the number of comparisons = 6]; therefore only *p* values <0.008 were considered significant in these conditions. For comparisons between 2 experimental conditions, *p* values <0.05 were considered significant. Differences in the cumulative distributions were assessed using the Kolmogorov-Smirnov (KS) test.

## Results

The aim of this work was to determine how the onset of visual experience regulates excitatory and inhibitory drive across cortical layers in the rat primary visual cortex (V1). We straddled the period from eye opening (P14) to the peak of the critical period for visual cortical plasticity (P30; Fagiolini et al., 1994) and quantified the distribution of two populations of inhibitory neurons, parvalbumin expressing (PV^+^) and somatostatin expressing (SST^+^). In addition, we measured excitatory and inhibitory synaptic drive onto pyramidal neurons grouped by laminar location. To assess how the time of eye opening contributes to the maturation of excitatory and inhibitory inputs onto V1 pyramidal neurons, we used two different manipulations to alter the onset of visual drive: bilateral delayed eye opening by binocular eyelid suture (bDEO) and unilateral delayed eye opening by monocular eyelid suture (mDEO).

### Age-dependent distribution of PV-expressing neurons in rat V1

Before assessing the effects of eye opening timing on the V1 cortical circuit, we needed to establish the pattern of maturation of excitatory and inhibitory synaptic drive in naive animals. Several studies showed that inhibitory synapses undergo a process of maturation during a developmental window that occurs between eye opening and the peak of the critical period for visual cortical plasticity (Kirkwood et al., 1995; Hensch et al., 1998; Rozas et al., 2001; Chattopadhyaya et al., 2004; Heinen et al., 2004). Many aspects of this process have been analyzed by quantifying some of the physiologic properties of inhibitory neurons (Lazarus and Huang, 2011) and inhibitory synaptic transmission in superficial layers (Rozas et al., 2001; Morales et al., 2002; Maffei et al., 2006, 2010). However, many issues remain unresolved. We asked whether the two best-studied populations of inhibitory neurons in V1, parvalbumin- and somatostatin-expressing (PV^+^ and SST^+^) neurons, are similarly distributed across cortical layers from the time of eye opening. To address this, we used immunohistochemistry and confocal microscopy and quantified number and location of PV^+^ and SST^+^ inhibitory neurons in the monocular region of V1 (V1m; Fig. 1*A*). We used VGluT2 staining, which labels thalamocortical axons, and fluorescent Nissl to identify cortical layers (Fig. 1*B*). Immunostaining for PV and SST was performed on fixed thin (50 μm) cortical slices obtained from rats of four postnatal age groups: P13–P14 (before eye opening), P17, P21 (onset of critical period in rat), and P30 (peak of critical period). In agreement with previous studies (Gonchar et al., 2007; Miyoshi et al., 2007), there was no colocalization of PV and SST in any age group (Fig. 1*C*). Analysis of the number of PV^+^ neurons revealed significant differences in their laminar distribution across age groups (Fig. 1*F*). As previously reported (del Rio et al., 1994; Gonchar et al., 2007) at P14, PV^+^ neurons were almost exclusively located in layer 5 (L5), whereas at P17, they were detectable in all layers. The density of PV^+^ neurons in each layer remained stable after P17 (Fig. 1*F*). (Percentage of PV^+^ neurons in L2/3: P14, 0.3 ± 0.2; P17, 3.9 ± 0.4; P21, 3.2 ± 0.4; P30, 2.7 ± 0.3; one-way ANOVA, *p* < 0.001; P14 vs. P17, *t* test *p* < 0.001; P14 vs. P21, *t* test *p* < 0.001; P14 vs. P30, *t* test *p* < 0.001; P17 vs. P21, *t* test *p* = 0.2; P17 vs. P30, *t* test *p* = 0.03; P21 vs. P30, *p* = 0.2; in L4: P14, 0.4 ± 0.4; P17, 5.9 ± 0.4; P21, 5.3 ± 0.6; P30, 3.9 ± 0.6; one-way ANOVA *p* < 0.001; P14 vs. P17, *t* test *p* < 0.001; P14 vs. P21, *t* test *p* < 0.001; P14 vs. P30, *t* test *p* = 0.003; P17 vs. P21, *t* test *p* = 0.45; P17 vs. P30 *t* test *p* = 0.05; P21 vs. P30 *t* test *p* = 0.17; in L5: P14, 3.2 ± 0.5; P17, 5.3 ± 0.5, P21, 4.5 ± 0.6; P30, 3.9 ± 0.6; one-way ANOVA *p* = 0.08; in L6: P14, 0.12 ± 0.16; P17, 2.3 ± 0.3; P21, 3.1 ± 0.5; P30, 2.8 ± 0.3; one-way ANOVA *p* = 0.009; P14 vs. P17, *t* test *p* < 0.001; P14 vs. P21, *t* test *p* = 0.002; P14 vs. P30, *t* test *p* = 0.001; P17 vs. P21, *t* test *p* = 0.36; P17 vs. P30, *t* test *p* = 0.29; P21 vs. P30, *p* = 0.85.)

In contrast, SST^+^ neurons showed a similar distribution across layers in all age groups (Fig. 1*G*). Percentage of SST^+^ neurons in L2/3: P14, 1.9 ± 0.3; P17, 2.1 ± 0.1; P21, 2.7 ± 0.2; P30, 2.6 ± 0.3; one-way ANOVA *p* = 0.08; in L4: P14, 2.3 ± 0.6; P17, 2.8 ± 0.3; P21, 2.8 ± 0.3; P30, 2.9 ± 0.7; one-way ANOVA *p* = 0.15; in L5: P14, 3.9 ± 0.8; P17, 3.2 ± 0.2; P21, 2.8 ± 0.3; P30, 2.7 ± 0.4; one-way ANOVA *p* = 0.09; in L6: P14, 2.2 ± 0.5; P17, 2.3 ± 0.2; P21, 3.1 ± 0.3; P30, 2.5 ± 0.3; one-way ANOVA *p* = 0.1. Overall, our results are consistent with previous findings from mouse V1 (Gonchar et al., 2007), showing that the density and laminar distribution of SST^+^ and PV^+^ neurons in V1 is differentially regulated during postnatal development. Although Gonchar et al. (2007) reported a progressive increase in SST^+^ neurons in the mouse after eye opening, our results indicate that in rat V1 they are similarly distributed across all cortical layers and their number is not affected by postnatal development. We also observed similarities and differences with the results reported by Gonchar et al. (2007) in regard to PV^+^ neurons. In both studies, PV^+^ neurons are found almost exclusively in L5 in the earliest age group. However, although Gonchar and collaborators report a progressive increase in PV^+^ neurons in L5 and other layers, we observed that in L5 the number of PV^+^ neurons remains stable across age groups. In all other layers, the number of PV^+^ neurons increases significantly and rapidly after eye opening. Our results suggest that in our preparation, SST^+^ neurons contribute significantly to the inhibitory drive onto pyramidal neurons in all layers of V1 from eye opening, while the maturation of PV^+^ neurons outside of L5 continues after the onset of vision.

### Laminar difference in synaptic drive during postnatal development

PV^+^ neurons provide a powerful source of perisomatic inhibition (Somogyi et al., 1983; Kawaguchi and Kubota, 1998). In view of the data in Fig. 1, we tested the hypothesis that changes in the number of PV^+^ neurons may underlie developmental shifts in the balance between excitatory and inhibitory synaptic drive onto pyramidal neurons located in each cortical layer.

**Figure 2.**
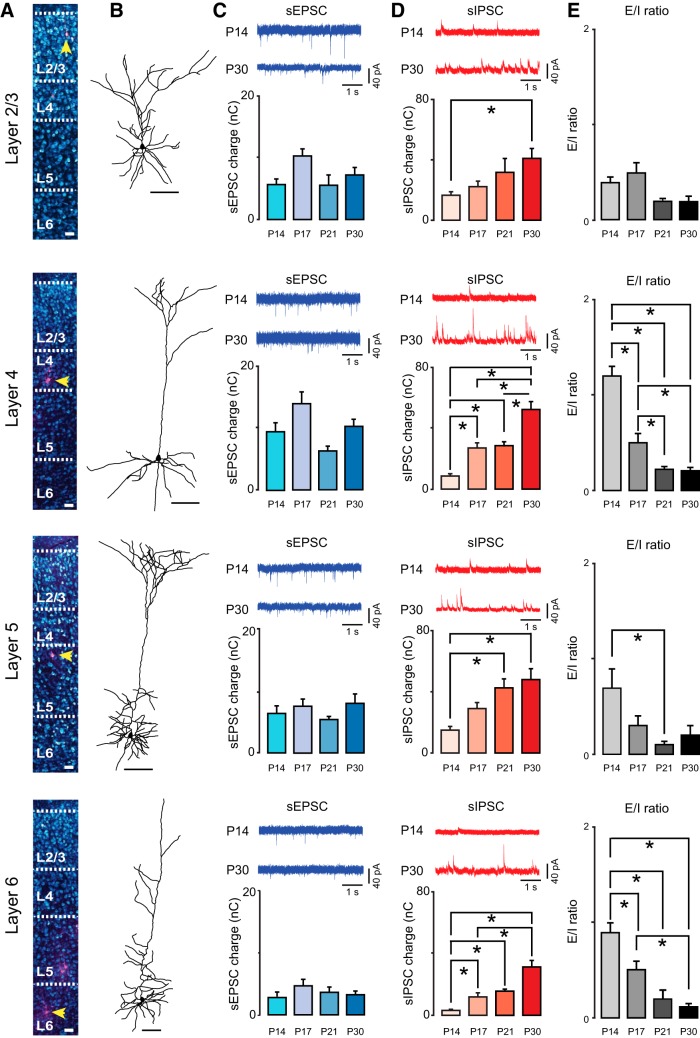
Laminar differences in excitatory and inhibitory synaptic drive. ***A***, From top to bottom: confocal images showing pyramidal neurons located respectively in L2/3, L4, L5, and L6. Yellow arrowheads indicate recorded neurons within the corresponding layer. It should be noted that multiple neurons were simultaneously recorded from different layers. Scale bars: 40 µm. ***B***, After confocal imaging, slices were unmounted and developed with DAB to reconstruct recorded neurons using the software Neurolucida on a bright-field microscope. We included in the analysis only data obtained from neurons with pyramidal cell morphology and intact apical dendrite. Scale bar: 100 µm. ***C***, From top to bottom: (upper panel, blue traces) representative traces of sEPSC recorded in L2/3, L4, L5, and L6 at P14 and P30; (lower panel, blue bar plots) average sEPSC charge quantified at P14, P17, P21, and P30. ***D***, From top to bottom: (upper panel, red traces) representative traces of sIPSC recorded in L2/3, L4, L5, and L6 at P14 and P30; (lower panel, red bar plots) average sIPSC charge quantified at P14, P17, P21, and P30. ***E***, Average excitatory/inhibitory (E/I ratio) ratio across layers in four postnatal age groups. Data are presented as mean ± standard error; asterisks indicate significant differences. Experimental values and statistics are reported in Tables 1 and 2.

We used voltage clamp recordings from visually identified pyramidal neurons in acute coronal slices containing the monocular region of V1 (V1m; Fig. 2*A*, *B*; Zilles et al., 1984; Reid and Juraska, 1991) and recorded spontaneous excitatory postsynaptic currents (sEPSCs, Fig. 2*C*) and spontaneous inhibitory postsynaptic currents (sIPSCs, Fig. 2*D*) in four postnatal age groups (P14, P17, P21, P30). The morphology and laminar location of all recorded neurons included in the analysis were confirmed *post hoc* with histologic procedures (Fig. 2*A*, *B*).

The overall excitatory and inhibitory synaptic drive onto pyramidal neurons was quantified as the synaptic charge flowing through the membrane over a 100 s time period. Excitatory charge was quantified from voltage clamp recordings obtained while holding neurons at –50 mV, the calculated reversal potential for chloride in our recording conditions; inhibitory charge was measured from currents recorded while holding neurons at 10 mV, the expected reversal potential for AMPA and NMDA receptor-mediated currents (Maffei et al., 2004; Maffei and Turrigiano, 2008). The excitatory charge onto pyramidal neurons in all layers of V1 did not change significantly in the four age groups (Fig. 2*C*; Table 1), suggesting that spontaneous excitatory drive onto pyramidal neurons is stable across the developmental window under study.

**Table 1.** Developmental changes in excitatory charge, sEPSC frequency, and sEPSC amplitude. ***A***, Multiple comparisons across ages and KW-ANOVA.

Layer	Charge (nC)					Frequency (Hz)					Amplitude (pA)				
	P14	P17	P21	P30	KW-ANOVA	P14	P17	P21	P30	KW-ANOVA	P14	P17	P21	P30	KW-ANOVA
L2/3	5.8 ± 0.8	10.4 ± 1.9	5.6 ± 1.5	7.4 ± 1.1	*p* = 0.2	2.3 ± 0.2	3.8 ± 0.6	2.9 ± 0.6	4.3 ± 0.5	*p = 0.007	21.6 ± 1.2	22.2 ± 2.1	16.7 ± 1.8	16.7 ± 1.7	*p = 0.02
L4	9.4 ± 1.5	13.8 ± 2.1	6.3 ± 0.7	10.2 ± 1.1	*p* = 0.06	3.6 ± 0.4	5.6 ± 0.7	3.3 ± 0.5	5.8 ± 0.5	*p = 0.005	21.2 ± 1.4	19.3 ± 1.2	18.2 ± 1.0	18.3 ± 1.7	*p* = 0.2
L5	6.4 ± 1.1	7.6 ± 1.1	5.4 ± 0.5	8.0 ± 1.5	*p* = 0.4	2.5 ± 0.5	2.9 ± 0.4	2.9 ± 0.5	3.7 ± 0.5	*p* = 0.3	21.1 ± 1.4	21.0 ± 1.8	19.7 ± 1.5	18.6 ± 1.6	*p* = 0.7
L6	2.9 ± 0.5	4.8 ± 1.0	3.6 ± 0.9	3.2 ± 0.5	*p* = 0.3	1.2 ± 0.3	2.2 ± 0.5	1.7 ± 0.4	2.1 ± 0.3	*p* = 0.3	19.9 ± 1.2	19.9 ± 1.1	20.0 ± 1.1	14.2 ± 0.7	*p = 0.002

Results are expressed as mean ± SEM.

**Table:** *B*, MW and KS tests.

Comparison	MW test				KS test			
	L2/3		L4 Frequency	L6 Amplitude	L2/3 Amplitude	L4 Amplitude	L5 Amplitude	L6 Amplitude
	Frequency	Amplitude						
P14 vs. P17	*p* = 0.04	*p* = 0.9	*p* = 0.02	*p* = 0.8	*p* = 0.3	*p < 0.001	*p = 0.0014	*p < 0.001
P14 vs. P21	*p* = 0.5	*p* = 0.06	*p* = 0.5	*p* = 0.6	*p < 0.001	*p < 0.001	*p < 0.001	*p < 0.001
P14 vs. P30	*p < 0.001	*p* = 0.02	*p = 0.005	*p = 0.002	*p < 0.001	*p < 0.001	*p < 0.001	*p < 0.001
P17 vs. P30	*p* = 0.4	*p* = 0.03	*p* = 0.7	*p = 0.002	*p < 0.001	*p < 0.001	*p < 0.001	*p < 0.001
P21 vs. P30	*p* = 0.03	*p* = 0.7	*p = 0.004	*p = 0.001	*p < 0.001	*p < 0.001	*p < 0.001	*p < 0.001
P17 vs. P21	*p* = 0.3	*p* = 0.09	*p* = 0.03	*p* = 0.9	*p* < 0.001	*p < 0.001	*p < 0.001	*p = 0.010

*Statistically significant. Only *p* values <0.008 are considered significant owing to Bonferroni correction for multiple comparisons.

In contrast, spontaneous inhibitory charge significantly increased in all layers between P14 and P30 (Fig. 2*D*; Table 2), suggesting that inhibitory circuits across all layers undergo a process of maturation during the postnatal window under study. The largest increases in inhibition were observed in the primary thalamorecipient layers, with a 6-fold increase in inhibitory drive in L4 and a 9-fold increase in L6. Although inhibitory drive was also significantly increased in L2/3 and L5, the size of this increase was relatively smaller in magnitude: 2.5-fold for L2/3 and 3-fold for L5. The developmental increase in inhibitory drive in the absence of significant changes in excitatory charge resulted in a progressive shift of the E/I balance toward inhibition in all layers (Fig. 2*E*).

**Table 2.** Developmental changes in inhibitory charge, sIPSC frequency, and sIPSC amplitude across age. ***A***, Multiple comparisons across ages and KW-ANOVA.

Layer	Charge (nC)					Frequency (Hz)					Amplitude (pA)				
	P14	P17	P21	P30	KW-ANOVA	P14	P17	P21	P30	KW-ANOVA	P14	P17	P21	P30	KW-ANOVA
L2/3	16.4 ± 2.4	22.3 ± 3.3	31.6 ± 9.3	40.7 ± 6.9	*p = 0.002	1.5 ± 0.2	2.7 ± 0.4	3.1 ± 0.5	4.9 ± 0.4	*p < 0.001	33.6 ± 1.5	29.8 ± 1.5	30.9 ± 3.9	27.0 ± 2.4	*p* = 0.07
L4	9.0 ± 1.7	27.5 ± 3.4	28.8 ± 2.4	52.3 ± 5.8	*p < 0.001	1.1 ± 0.2	3.4 ± 0.4	3.2 ± 0.4	5.6 ± 0.3	*p < 0.001	29.7 ± 1.9	31.2 ± 2.1	31.4 ± 2.0	37.0 ± 2.1	*p* = 0.2
L5	15.1 ± 2.8	29.3 ± 3.7	42.8 ± 5.8	48.3 ± 7.2	*p < 0.001	2.2 ± 0.5	3.4 ± 0.4	4.5 ± 0.5	5.1 ± 0.4	*p = 0.002	27.4 ± 1	29.6 ± 2.7	31.2 ± 2.5	32.4 ± 2.7	*p* = 0.7
L6	3.6 ± 0.6	12.5 ± 2.5	16.4 ± 0.7	31.7 ± 4.6	*p < 0.001	0.5 ± 0.1	1.7 ± 0.4	1.9 ± 0.3	3.5 ± 0.4	*p < 0.001	25.6 ± 2.0	29.2 ± 1.5	29.1 ± 1.7	30.3 ± 4.1	*p* = 0.4

Results are expressed as Mean ± SEM.

**Table:** *B*, MW and KS tests.

Comparison	MW test								KS test			
	L2/3		L4		L5		L6		L2/3 Amplitude	L4 Amplitude	L5 Amplitude	L6 Amplitude
	Charge	Frequency	Charge	Frequency	Charge	Frequency	Charge	Frequency				
P14 vs. P17	*p* = 0.2	*p* = 0.01	*p < 0.001	*p < 0.001	*p* = 0.01	*p* = 0.09	*p < 0.001	*p < 0.001	*p < 0.001	*p < 0.001	*p < 0.001	*p < 0.001
P14 vs. P21	*p* = 0.07	*p* = 0.01	*p < 0.001	*p < 0.001	*p < 0.001	*p* = 0.01	*p = 0.007	*p < 0.001	*p < 0.001	*p* = 0.035	*p < 0.001	*p < 0.001
P14 vs. P30	*p < 0.001	*p < 0.001	*p < 0.001	*p < 0.001	*p = 0.001	*p = 0.002	*p < 0.001	*p < 0.001	*p < 0.001	*p < 0.001	*p < 0.001	*p < 0.001
P17 vs. P30	*p* = 0.01	*p = 0.001	*p < 0.001	*p < 0.001	*p* = 0.04	*p* = 0.01	*p = 0.002	*p = 0.002	*p < 0.001	*p < 0.001	*p < 0.001	*p < 0.001
P21 vs. P30	*p* = 0.1	*p* = 0.02	*p = 0.001	*p < 0.001	*p* = 0.6	*p* = 0.3	*p* = 0.01	*p* = 0.02	*p* = 0.058	*p < 0.001	*p < 0.001	*p < 0.001
P17 vs. P21	*p* = 0.5	*p* = 0.6	*p* = 0.9	*p* = 0.8	*p* = 0.08	*p* = 0.1	*p* = 0.3	*p* = 0.4	*p < 0.001	*p = 0.002	*p < 0.001	*p* = 0.3

*Statistically significant. Only *p* values <0.008 are considered significant due to Bonferroni correction for multiple comparisons.

**Figure 3.**
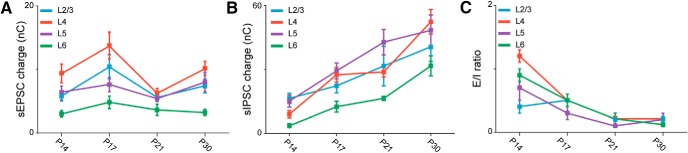
Laminar differences in synaptic charge and E/I balance. ***A***, Developmental time course of excitatory charge in L2/3 (blue), L4 (red), L5 (purple), and L6 (green). ***B***, Time course of inhibitory charge in the different layers. ***C***, Time course of the E/I ratio of the charges compared across layers. Statistical analysis for these data are provided in Table 3.

In addition to this overall effect on synaptic charge, interesting laminar differences in the E/I ratio were observed. Compared with other layers, the inputs onto L4 pyramidal neurons showed the largest difference between excitatory charge and inhibitory charge at P14. Overall, at each time point analyzed, L6 received the least excitation and inhibition compared with all other layers (Fig. 3*A*, *B*; Table 3). Comparisons of E/I ratios across layers indicated that at eye opening, synaptic drive onto L2/3 and L5 was already significantly inhibition-shifted (below 1), whereas it was balanced (close to 1) in L6 and excitation-shifted (above 1) in L4. In L4, dominant excitation was due to larger excitatory charge and significantly lower inhibitory charge compared with other layers (Fig. 3*B*, *C*). By P30, as inhibitory drive progressively increased, the E/I ratio reached similar values in all layers (Fig. 3*C*). (E/I ratio in L2/3: P14, 0.4 ± 0.06, *n* = 13; P17, 0.5 ± 0.1, *n* = 11; P21, 0.20 ± 0.03, *n* = 10; P30, 0.2 ± 0.05, *n* = 12; KW ANOVA: *p* = 0.007; MW test: P14 vs. P17, *p* = 0.5; P14 vs. P21, *p* = 0.03; P14 vs. P30, *p* = 0.02; P17 vs. P21, *p* = 0.01; P17 vs. P30, *p* = 0.009; P21 vs. P30, *p* = 0.9; in L4: P14, 1.2 ± 0.1, *n* = 14; P17, 0.5 ± 0.1, *n* = 14; P21, 0.22 ± 0.03, *n* = 10; P30, 0.21 ± 0.03, *n* = 11; KW ANOVA: *p* < 0.001; MW test: P14 vs. P17, *p* < 0.001; P14 vs. P21, *p* < 0.001; P14 vs. P30, *p* < 0.001; P17 vs. P21, *p* = 0.002; P17 vs. P30, *p* = 0.001; P21 vs. P30, *p* = 0.9; in L5: P14, 0.7 ± 0.2, *n* = 10; P17, 0.3 ± 0.1, *n* = 14; P21, 0.10 ± 0.03, *n* = 10; P30, 0.2 ± 0.1, *n* = 10; KW ANOVA: *p* = 0.01; MW test: P14 vs. P17, *p* = 0.08: P14 vs. P21, *p* = 0.002; P14 vs. P30, *p* = 0.02; P17 vs. P21, *p* = 0.06; P17 vs. P30, *p* = 0.1; P21 vs. P30, *p* = 0.80; in L6: P14, 0.9 ± 0.1, *n* = 13; P17, 0.5 ± 0.1, *n* = 13; P21, 0.2 ± 0.1, *n* = 10; P30, 0.12 ± 0.02, *n* = 11; KW ANOVA: *p* < 0.001; MW test: P14 vs. P17, *p* = 0.004: P14 vs. P21, *p* < 0.001: P14 vs. P30, *p* < 0.001; P17 vs. P21, *p* = 0.07; P17 vs. P30, *p* = 0.001; P21 vs. P30, *p* = 0.1). Only *p* values <0.008 are considered significant owing to correction for multiple comparisons. In summary, these data show that from the time of eye opening to the peak of the critical period, synaptic drive onto pyramidal neurons in V1m differed significantly across layers. In L2/3 and L5, inhibition was dominant in all age groups, whereas it transitioned from dominant excitation to dominant inhibition in L4 and from balanced to inhibition-shifted in L6. The distinct starting points converge on a comparable inhibition-dominated state over the course of the 2 wks after eye opening (Fig. 3*C*).

**Table 3.** Multicomparisons of excitatory and inhibitory synaptic transmission across layers.

Comparison	sEPSC charge	sEPSC frequency	sEPSC amplitude	sIPSC charge	sIPSC frequency	sIPSC amplitude	E/I ratio
P14							
KW-ANOVA	*p < 0.001	*p < 0.001	*p* = 0.8	*p < 0.001	*p < 0.001	*p = 0.007	*p < 0.001
M-W *U*							
L2/3 vs. L4	*p* = 0.03	*p = 0.007		*p* = 0.01	*p* = 0.07	*p* = 0.1	*p < 0.001
L2/3 vs. L5	*p* = 0.7	*p* = 0.9		*p* = 0.6	*p* = 0.4	*p = 0.005	*p* = 0.5
L2/3 vs. L6	*p* = 0.01	*p* = 0.01		*p < 0.001	*p < 0.001	*p = 0.004	*p = 0.001
L4 vs. L5	*p* = 0.08	*p* = 0.09		*p* = 0.07	*p* = 0.06	*p* = 0.3	*p* = 0.01
L4 vs. L6	*p < 0.001	*p < 0.001		*p = 0.005	*p = 0.003	*p* = 0.1	*p* = 0.07
L5 vs. L6	*p* = 0.02	*p* = 0.04		*p < 0.001	*p < 0.001	*p* = 0.2	*p* = 0.1
P17							
KW-ANOVA	*p = 0.01	*p = 0.001	*p* = 0.8	*p = 0.001	*p = 0.005	*p* = 0.8	*p* = 0.07
M-W *U*							
L2/3 vs. L4	*p* = 0.2	*p* = 0.09		*p* = 0.3	*p* = 0.2		
L2/3 vs. L5	*p* = 0.6	*p* = 0.2		*p* = 0.2	*p* = 0.4		
L2/3 vs. L6	*p* = 0.03	*p* = 0.02		*p* = 0.01	*p* = 0.04		
L4 vs. L5	*p* = 0.08	*p = 0.005		*p* = 0.8	*p* = 0.7		
L4 vs. L6	*p = 0.005	*p < 0.001		*p = 0.001	*p = 0.002		
L5 vs. L6	*p* = 0.1	*p* = 0.1		*p = 0.001	*p = 0.003		
P21							
KW-ANOVA	*p* = 0.1	*p* = 0.2	*p* = 0.5	*p = 0.002	*p = 0.009	*p* = 0.8	*p* = 0.4
M-W *U*							
L2/3 vs. L4				*p* = 0.6	*p* = 0.6		
L2/3 vs. L5				*p* = 0.1	*p* = 0.08		
L2/3 vs. L6				*p* = 0.1	*p* = 0.3		
L4 vs. L5				*p* = 0.1	*p* = 0.08		
L4 vs. L6				*p < 0.001	*p* = 0.02		
L5 vs. L6				*p < 0.001	*p < 0.001		
P30							
KW-ANOVA	*p < 0.001	*p < 0.001	*p* = 0.1	*p* = 0.07	*p = 0.01	*p = 0.03	*p* = 0.2
M-W *U*							
L2/3 vs. L4	*p* = 0.1	*p* = 0.04			*p* = 0.3	*p = 0.004	
L2/3 vs. L5	*p* = 0.8	*p* = 0.5			*p* = 0.7	*p* = 0.1	
L2/3 vs. L6	*p = 0.004	*p = 0.001			*p* = 0.03	*p* = 0.8	
L4 vs. L5	*p* = 0.2	*p* = 0.01			*p* = 0.7	*p* = 0.1	
L4 vs. L6	*p < 0.001	*p < 0.001			*p = 0.004	*p* = 0.07	
L5 vs. L6	*p = 0.007	*p* = 0.02			*p* = 0.02	*p* = 0.4	

Development: sEPSCs and sIPSCs; multiple comparisons across layers. *Statistically significant. Only *p* values <0.008 are considered significant due to Bonferroni correction for multiple comparisons.

### Layer-specific mechanisms of maturation for synaptic transmission

**Figure 4.**
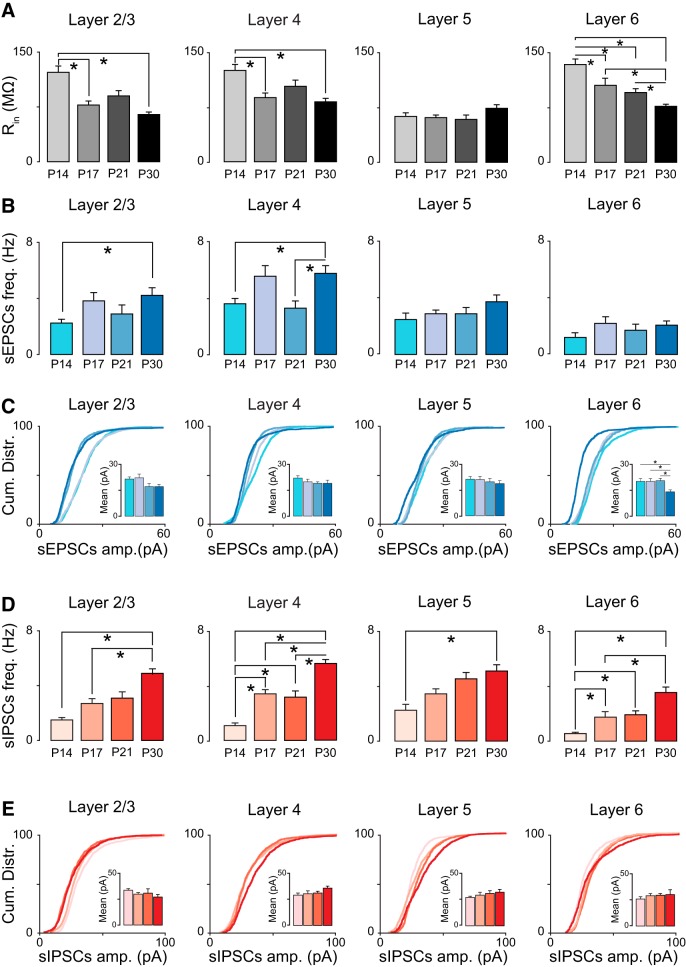
Maturation of spontaneous excitatory and inhibitory inputs in V1. ***A***, Average input resistance (R_in_) measured from V1m pyramidal neurons in L2/3, L4, L5, and L6 and in the different age groups (P14, P17, P21, P30). ***B***, From left to right: average sEPSC frequency measured from pyramidal neurons grouped by layer and age group. ***C***, From left to right: cumulative distributions of sEPSC amplitude from L2/3, L4, L5, and L6 pyramidal neurons in the four age groups. Small bar plots represent average sEPSC amplitudes. ***D***, From left to right: average sIPSC frequency measured by layer and age group. ***E***, From left to right: cumulative distributions of sIPSC amplitude from L2/3, L4, L5, and L6 pyramidal neurons in the four age groups. Small bar plots represent average sIPSC amplitudes. 100 events/neuron were included in the cumulative plots. Data are mean ± SEM. Values and statistics are reported in Tables 1 and 2.

Synaptic charge is a compound measurement that depends on the amplitude and frequency of synaptic currents. Developmental changes in input resistance could also affect synaptic responses. We assessed whether the resting input resistance (R_in_) of pyramidal neurons changes during development and found a progressive decrease in L2/3, L4, and L6, but not L5 (Fig. 4*A*). (R_in_ (MΩ) in L2/3: P14, 123 ± 8, *n* = 14; P17, 79 ± 4, *n* = 11; P21, 90 ± 7, *n* = 10; P30, 65 ± 3, *n* = 12; KW ANOVA: *p* < 0.001; MW test: P14 vs. P17, *p* < 0.001; P14 vs. P21, *p* = 0.03; P14 vs. P30, *p* < 0.001; P17 vs. P21, *p* = 0.09; P17 vs. P30, *p* = 0.01; P21 vs. P30, *p* = 0.009; in L4: P14, 126 ± 7, *n* = 14; P17, 89 ± 5, *n* = 14; P21, 104 ± 8, *n* = 10; P30, 82 ± 5, *n* = 11; KW ANOVA: *p* < 0.001; MW test: P14 vs. P17, *p* < 0.001; P14 vs. P21, *p* = 0.08; P14 vs. P30, *p* < 0.001; P17 vs. P21, *p* = 0.25; P17 vs. P30, *p* = 0.29; P21 vs. P30, *p* = 0.06; in L5: P14, 62 ± 5, *n* = 10; P17, 61 ± 3, *n* = 14; P21, 59 ± 5, *n* = 10; P30, 73 ± 5, *n* = 10; KW ANOVA: *p* = 0.1; in L6: P14, 134 ± 7, *n* = 13; P17, 105 ± 10; P21, 96 ± 5, *n* = 10; P30, 76 ± 3, *n* = 11; KW ANOVA: *p* < 0.001; MW test: P14 vs. P17, *p* = 0.005; P14 vs. P21, *p* = 0.001; P14 vs. P30, *p* < 0.001; P17 vs. P21, *p* = 0.8; P17 vs. P30, *p* = 0.004; P21 vs. P30, *p* < 0.001). Only *p* values <0.008 are considered significant owing to the correction for multiple comparisons). We then quantified frequency and amplitude of sEPSCs and sIPSCs across cortical layers between P14 and P30 (Fig. 4). Despite the absence of change in excitatory charge, analysis of sEPSC frequency and amplitude shows that this did not imply lack of developmental regulation of excitatory synaptic transmission. The frequency of sEPSCs progressively and significantly increased in L2/3 and L4, whereas it did not change significantly in L5 and L6 (Fig. 4*B*; Table 1), indicating a selective developmental regulation of sEPSC frequency in superficial layers of V1.

**Figure 5.**
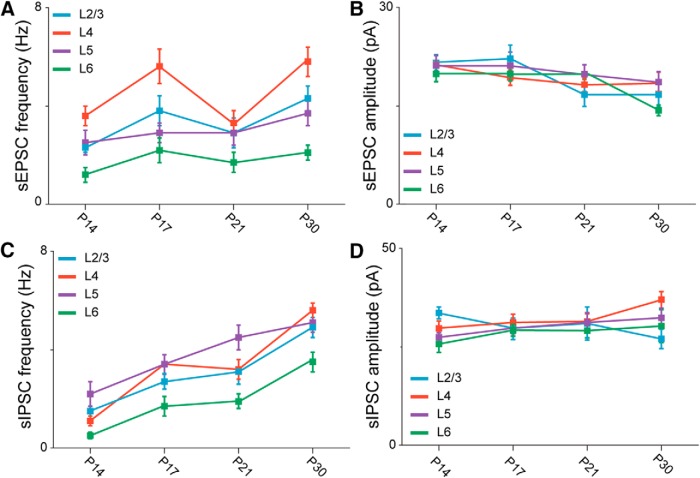
Laminar differences in excitatory and inhibitory synaptic transmission. ***A***, ***B***, Time course of sEPSC frequency (***A***) and amplitude (***B***) compared across layers. L2/3: blue; L4: red; L5: purple; L6: green. ***C***, ***D***, sIPSC frequency (***C***) and amplitude (***D***) plotted by layer and age group. The statistical analyses for the data are reported in Table 3.

The average sEPSC amplitude showed a significant decrease only in L6 (Fig. 4*C*; Table 1). However, analysis of the cumulative distribution of sEPSC amplitudes across ages and laminae unveiled a progressive and significant shift toward a predominance of small sEPSCs in all layers (Fig. 4*C*; see KS test in Table 1). These data suggest the engagement of different mechanisms for postnatal maturation of excitatory synaptic transmission in superficial and deep layers of V1m. In L2/3 and L4, there was a significant increase in sEPSC frequency and a shift in the distribution, in the absence of changes in the average sEPSC amplitude. The layer specificity of the developmental changes in sEPSC frequency and amplitude is better represented in Fig. 5*A*, *B*, where amplitude and frequency of synaptic events are compared across layers (Table 3). Taken together, the data suggest that the postnatal maturation of excitatory synaptic transmission in L2/3, L4, and L5 is not due to a decrease in R_in_ and point to the involvement of presynaptic mechanisms for the regulation of excitatory synaptic drive onto L2/3 and L4 pyramidal neurons. In L6, the sEPSC frequency remains unchanged, but both the average sEPSC amplitude and the distribution of sEPSC amplitude showed significant decreases, suggesting the engagement of postsynaptic mechanisms and a likely influence of R_in_.

Unlike excitatory drive, which did not show significant change during the time window analyzed, GABAergic inhibitory charge significantly increased in all layers after eye opening (Fig. 2*D*; Table 2). To assess whether this increase may be expressed via presynaptic or postsynaptic mechanisms, we quantified frequency and amplitude of sIPSCs and observed a progressive and significant increase in sIPSC frequency in all layers in the developmental window under study (Fig. 4*D*; Table 2).

The average amplitude of sIPSCs remained stable; however, the distribution of sIPSC amplitudes showed a significant shift toward a predominance of small events in L2/3 from P14 to P30 (Fig. 4*E*; see KS test in Table 2), while all other layers showed a shift in the distribution of sIPSC amplitudes toward larger events (Fig. 4*D*; Table 2). The laminar specificity of these changes is best represented in Fig. 5*C*, *D*, where the time course of the changes in sIPSC frequency and amplitude is compared across layers (Table 3). The shift in distribution of sIPSC amplitudes in the absence of changes in the average sIPSC amplitude suggests that the developmental increase in inhibitory drive is independent of the changes in R_in_ in L4–L6, whereas a contribution of R_in_ may explain the decrease sIPSC amplitude in the face of increased sIPSC frequency in L2/3. In summary, the maturation of inhibitory drive in all cortical layers is likely due to a progressive increase in presynaptic function from the time of eye opening to the peak of the critical period, resulting in larger inhibitory charge onto all V1 pyramidal neurons.

### Effects of binocular delayed eye opening on synaptic transmission in V1m

It is well accepted that visual experience shapes the maturation of V1. Most studies to date focused on the effects of altered visual experience during the critical period for ocular dominance plasticity. Much less is known about how the time of eye opening may affect the maturation of V1 circuits.

**Figure 6.**
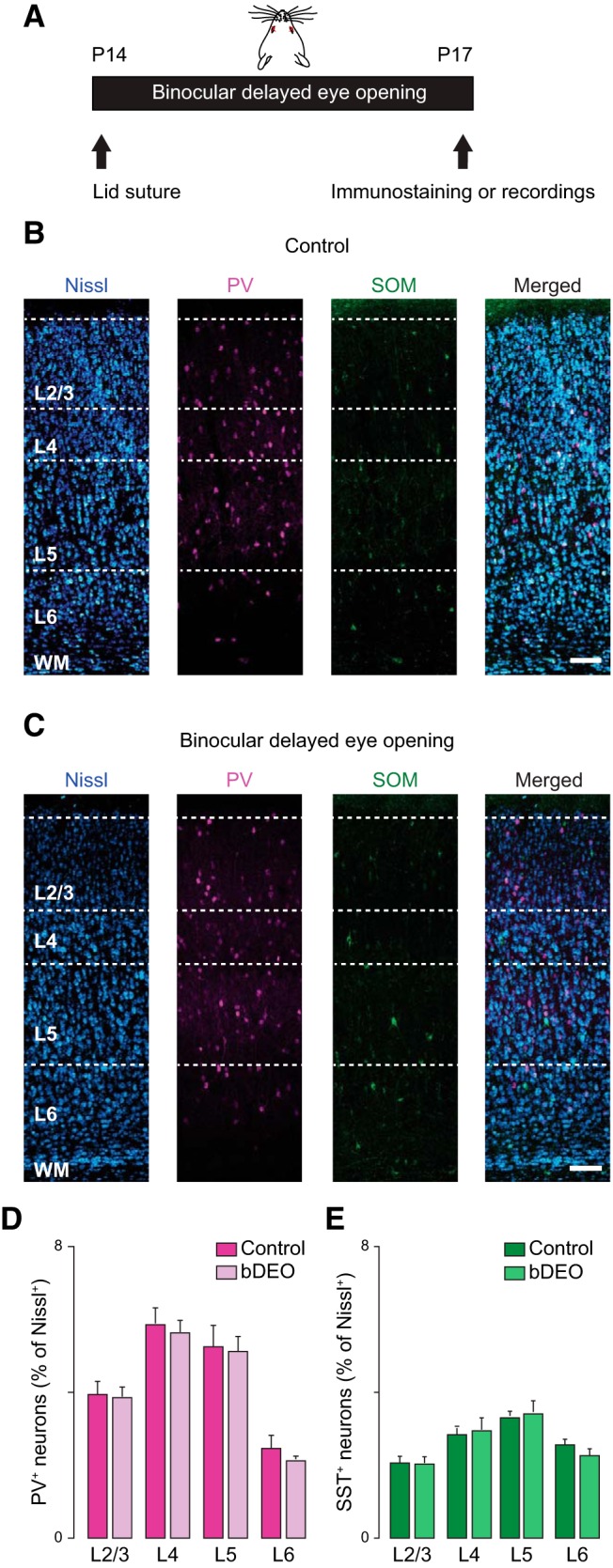
bDEO does not affect PV^+^ and SST^+^ neurons number in rat V1m. ***A***, Diagram of binocular delayed eye opening (bDEO): both eyes were sutured shut before eye opening and kept closed until P17. ***B***, From left to right: confocal images taken from control rats at P17 (Control) showing Nissl staining (blue), PV^+^ (magenta), and SST^+^ (green) immunostaining and merge. Scale bar: 125 µm. ***C***, From left to right: confocal images taken from P17 bDEO rats showing Nissl staining (blue), PV^+^ (magenta), and SST^+^ (green) immunostaining and merge. Scale bar: 125 µm. ***D***, Quantification of the percentage of PV^+^ interneurons in P17 control and P17 bDEO rats. ***E***, Quantification of the SST^+^ interneurons from P17 control and P17 bDEO rats. 10 coronal slices from 4 rats were used to quantify PV and SST expression. Data are mean ± SEM.

As the number of neurons expressing PV increased significantly between eye opening and P17 (Fig. 1), we asked whether this process may depend on the time of eye opening. To address this question, we delayed eye opening with binocular eyelid suture (bDEO) for 3 d (Fig. 6*A*) and compared the proportion of PV^+^ neurons in bDEO (bDEO P17) rats and age-matched nondeprived littermates (Control P17). Thin, fixed slices obtained from the two groups of rats were immunostained with antibodies against PV and SST (Fig. 6*B*, *C*), and the percentage of PV^+^ and SST^+^ neurons was quantified from images obtained with confocal microscopy. There was no significant difference in the number of PV^+^ and SST^+^ neurons between bDEO and control rats, indicating that the increase in PV^+^ neurons at P17 is not dependent on the time of eye opening, and that the percentage of both neuron groups is not sensitive to binocular delays in the onset of visual drive (Fig. 6*D*, *E*). Percentage of PV^+^ neurons in L2/3: Control P17, 3.8 ± 0.5; bDEO, 3.7 ± 0.1; unpaired *t* test, *p* = 0.8; in L4: Control P17, 5.9 ± 0.5; bDEO, 5.6 ± 0.4; unpaired *t* test, *p* = 0.6; in L5: Control P17, 5.3 ± 0.5; bDEO, 5.1 ± 0.4; unpaired *t* test, *p* = 0.2; in L6, Control P17, 2.5 ± 0.4; bDEO, 2.1 ± 0.1; unpaired *t* test, *p* = 0.5; in L2/3: Control P17, 2.1 ± 0.1; bDEO, 2.1 ± 0.1; unpaired *t* test, *p* = 0.8; in L4, Control P17, 2.6 ± 0.1; bDEO, 2.8 ± 0.3; unpaired *t* test, *p* = 0.7; in L5: Control P17, 3.2 ± 0.3; bDEO, 3.4 ± 0.4; unpaired *t* test, *p* = 0.9; in L6, Control P17, 2.5 ± 0.1; bDEO, 2.2 ± 0.2; *p* = 0.2.

**Figure 7.**
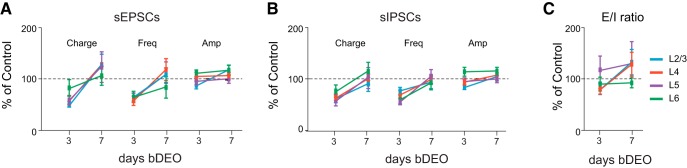
Effect of 3- and 7-d bDEO on synaptic transmission and E/I ratio. ***A***, Effect of bDEO on sEPSC charge, frequency, and amplitude plotted by layer and as percentage of control (represented by the dotted line). L2/3: blue; L4: red; L5: purple; L6: green. ***B***, Laminar changes in sIPSC charge, frequency, and amplitude induced by 3- or 7-d bDEO. ***C***, Laminar ratio of excitatory and inhibitory charge after 3- and 7-d bDEO. The statistical analyses for these data are reported in Tables 4 and 5.

We then assessed whether 3-d bDEO may have significant effects on synaptic transmission. Spontaneous EPSCs and sIPSCs were recorded from pyramidal neurons in slices prepared from V1m of bDEO and nondeprived age-matched littermates. 3-d bDEO significantly reduced excitatory charge in L2/3, L4, and L5 (Fig. 7*A*; Table 4, bDEO P17). No differences were observed in L6 (Fig. 7*A*; Table 4). When frequency and amplitude of sEPSCs were quantified, we observed a significant reduction in sEPSC frequency and no change in average amplitude in L2/3, L4, and L5 (Fig. 7*A*; Table 4). These data suggest that the bDEO affects excitatory synaptic transmission in L2/3–L5 by engaging presynaptic mechanisms, and that excitatory inputs onto L6 neurons are insensitive to 3-d bDEO.

**Table 4.** Changes in excitatory and inhibitory transmission after bDEO.

Layer	EPSC charge		EPSC frequency		EPSC amplitude		IPSC charge		IPSC frequency		IPSC amplitude		E/I ratio	
	bDEOP17	bDEOP21	bDEOP17	bDEOP21	bDEOP17	bDEOP21	bDEOP17	bDEOP21	bDEOP17	bDEOP21	bDEOP17	bDEOP21	bDEOP17	bDEOP21
L2/3	51 ± 5; *p = 0.01	126 ± 22; p = 0.5	64 ± 7; *p = 0.04	107 ± 14; p = 0.8	88 ± 7; p = 0.3	116 ± 10; p = 0.3	63 ± 9; *p = 0.03	90 ± 13; p = 0.8	77 ± 7; p = 0.1	94 ± 12; p = 0.8	84 ± 5; *p = 0.04	104 ± 7; p = 0.8	83.6 ± 12.1 p = 0.5	134.8 ± 24.8 p = 0.3
L4	60 ± 9; *p = 0.03	128 ± 25; p = 0.3	57 ± 9; *p = 0.01	118 ± 21; p = 0.6	105 ± 8; p = 0.6	106 ± 6; p = 0.3	64 ± 6; *p = 0.01	99 ± 10; p = 0.9	70 ± 8; *p = 0.02	99 ± 8; p = 0.9	94 ± 5; p = 0.5	107 ± 2; p = 0.2	83.0 ± 9.5p = 0.4	130.6 ± 23.4p = 0.3
L5	59 ± 8; *p = 0.03	122 ± 30; p = 0.5	63 ± 9; *p = 0.03	107 ± 23; p = 0.8	95 ± 7; p = 0.7	99 ± 11; p = 0.9	59 ± 10; *p = 0.02	101 ± 20; p = 0.9	56 ± 6; *p = 0.01	105 ± 13; p = 0.8	96 ± 7; p = 0.7	99 ± 7; p = 0.9	119.8 ± 26.9p = 0.5	131.4 ± 43.8p = 0.5
L6	82 ± 16; p = 0.5	106 ± 18; p = 0.8	65 ± 11; p = 0.2	84 ± 21; p = 0.6	111 ± 6; p = 0.2	117 ± 10; p = 0.1	77 ± 12; p = 0.3	114 ± 18; p = 0.1	60 ± 9; p = 0.07	93 ± 14; p = 0.4	114 ± 9; p = 0.1	116 ± 8; p = 0.1	91.9 ± 13.5; p = 0.7	94.3 ± 9.6; p = 0.8

Results are expressed as mean % change from control (age-matched littermates) ± SEM. *Statistically significant (*p* < 0.05) by *t* test.

The effects of bDEO were transient, as analysis of excitatory synaptic transmission onto pyramidal neurons in preparations from rats whose time of eye opening was delayed by 7 d showed no differences from recordings in age-matched littermates (Fig. 7*A*; Table 4, bDEO P21). Thus, excitatory synaptic transmission in L2/3, L4, and L5 is sensitive to a 3-d shift in the time of eye opening; however, if delays are protracted beyond 3 d, it appears that compensatory mechanisms are engaged to bring excitatory synaptic transmission back to control levels.

A similar pattern of changes was observed when quantifying the effects of bDEO on inhibitory synaptic transmission. There was a significant decrease in inhibitory charge in L2/3, L4, and L5 after 3-d bDEO, but all parameters returned to baseline if eye opening was delayed by 7 d (Fig. 7*B*; Table 4). Interestingly, in L4 and L5, the decrease in inhibitory charge was due to a significant decrease in sIPSC frequency (Fig. 7*B*; Table 4), whereas in L2/3, there was also a decrease in the average sIPSC amplitude (Fig. 7*B*; Table 4), indicating that the mechanisms for the reduction of inhibitory charge in L2/3 differ from those engaged in L4 and L5. The effect of 3-d bDEO on sIPSCs was not dependent on changes in R_in_, as the visual manipulation increased the R_in_ selectively in L2/3 and L4 pyramidal neurons. R_in_ (MΩ) in L2/3: Control, 79 ± 4, *n* = 11; bDEO P17, 100 ± 6, *n* = 13; unpaired *t* test, *p* = 0.01; in L4: Control, 89 ± 5, *n* = 14; bDEO P17, 114 ± 7, *n* = 13; unpaired *t* test, *p* = 0.006; in L5: Control, 61 ± 3, *n* = 14; bDEO P17, 59 ± 4, *n* = 12; unpaired *t* test, *p* = 0.1; in L6: Control, 105 ± 10, *n* = 13; bDEO P17, 125 ± 11, *n* = 14; unpaired *t* test, *p* = 0.2. Increased R_in_ is typically associated with an increase in the amplitude of synaptic responses, but we observed a reduction.

Excitatory and inhibitory charge were reduced proportionally in L2/3, L4, and L5; thus the E/I ratio was not affected by 3-d bDEO (L2/3: Control P17, 0.55 ± 0.14 (*n* = 11); bDEO P17, 0.46 ± 0.07 (*n* = 13); unpaired *t* test, *p* = 0.9. L4: Control P17, 0.54 ± 0.08 (*n* = 14); bDEO P17, 0.45 ± 0.05 (*n* = 13); unpaired *t* test, *p* = 0.4. L5: Control P17, 0.31 ± 0.06 (*n* = 14); bDEO P17, 0.37 ± 0.08 (*n* = 12); unpaired *t* test, *p* = 0.5. L6: Control P17, 0.46 ± 0.10 (*n* = 13); bDEO P17, 0.42 ± 0.06 (*n* = 14); unpaired *t* test, *p* = 0.7). No change in E/I ratio was observed for 7-d bDEO as well (L2/3: Control P21, 0.20 ± 0.03; bDEO P21, 0.27 ± 0.05; unpaired *t* test, *p* = 0.3. L4: Control P21, 0.22 ± 0.03; bDEO P21, 0.29 ± 0.05; unpaired *t* test, *p* = 0.3. L5: Control P21, 0.15 ± 0.03; bDEO P21, 0.20 ± 0.07; unpaired *t* test, *p* = 0.5. L6: Control P21, 0.22 ± 0.1; bDEO P21, 0.21 ± 0.02; unpaired *t* test, *p* = 0.8). The percentage change of the E/I ratio in all layers for bDEO P17 and bDEO P21 is represented in Fig. 7*C* (see statistical analysis in Table 4), and comparisons of the changes across layers are reported in Table 5. Altogether, our results show that the maturation of excitatory and inhibitory synaptic transmission in L2/3, L4, and L5, but not in L6, is sensitive to 3-d bDEO. Compensatory mechanisms appear to be engaged if the delay is protracted to 7 d, as all parameters return to control levels.

**Table 5.** Multiple comparisons of excitatory and inhibitory synaptic transmission after bDEO.

bDEO	sEPSC charge	sEPSC frequency	sEPSC amplitude	sIPSC charge	sIPSC frequency	sIPSC amplitude	E/I ratio
P17	*p* = 0.8	*p* = 0.9	*p* = 0.06	*p* = 0.7	*p* = 0.2	*p* = 0.08	*p* = 0.8
P21	*p* = 0.9	*p* = 0.6	*p* = 0.5	*p* = 0.6	*p* = 0.8	*p* = 0.6	*p* = 0.7

Multiple comparisons across layers by KW-ANOVA. Values for bDEO P17 and P21 are reported in Table 4.

### Laminar-specific effects of unilateral delayed eye opening in V1m

**Figure 8.**
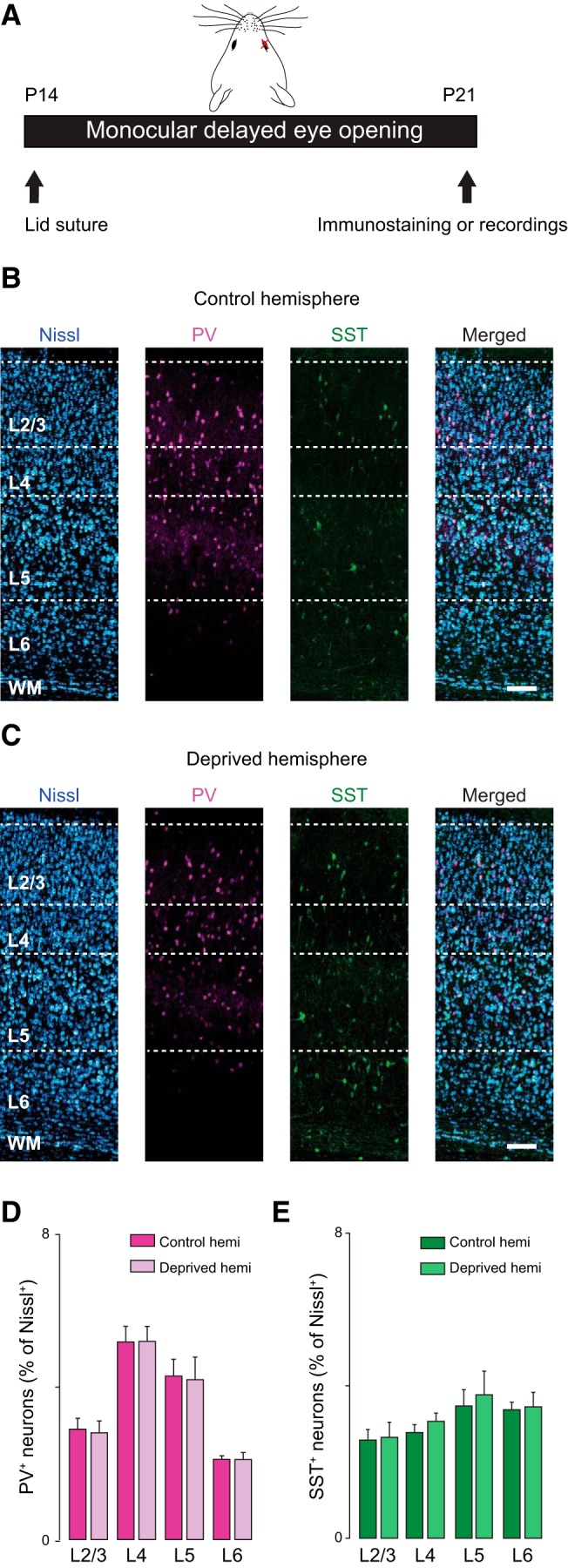
The number and distribution of PV^+^ and SST^+^ neurons is not altered by mDEO. ***A***, Diagram of mDEO: one eye was sutured shut right before eye opening (P13–P14) and kept closed until P21. ***B***, From left to right: confocal images taken from the control hemisphere (ipsilateral to the closed eye) showing Nissl (blue), PV^+^ (magenta), and SST^+^ (green) staining and merge. Scale bar: 125 µm. ***C***, From left to right: confocal images taken from the hemisphere contralateral to the closed eye (deprived) showing Nissl, PV^+^, and SST^+^ staining and merge. Scale bar: 125 µm. ***D***, Quantification of the percentage of PV^+^ interneurons in control and deprived hemispheres across V1 cortical layers (data obtained from 10 coronal slices from 4 rats). ***E***, Quantification of the percentage of SST^+^ interneurons in control and deprived hemispheres across V1 cortical layers (data were collected from 10 coronal slices from 4 rats). Data are mean ± SEM.

Delayed eye opening reduces visual drive uniformly to both eyes. However, in pathologic conditions such as unilateral cataracts, the onset of patterned vision can be mismatched. Previous work suggests that bilateral and unilateral delayed eye opening may have different effects on the circuit in V1 (Ellemberg et al., 1999, 2000, 2002). We therefore asked what would be the effect of delaying eye opening unilaterally [monocular delayed eye opening (mDEO)]. In two groups of rats, we delayed the opening of one eye for either 3 or 7 d with unilateral eyelid suture. The eyelid was sutured when the eyes were still closed, a day before the expected time of eye opening (P13–P14; Fig. 8*A*). Data were obtained from V1m of the hemisphere contralateral to the lid closure (mDEO) and compared with those obtained from V1m of the hemisphere ipsilateral to the closed eye (Control). We quantified the effect of mDEO on the number of PV^+^ and SST^+^ neurons, as well as on synaptic transmission onto pyramidal neurons.

Monocular DEO did not affect the number of PV^+^ and SST^+^ neurons in any layer of V1m, indicating that the developmental regulation of the distribution of these inhibitory neurons is independent of the time of eye opening (Fig. 8*D*, *E*). Percentage of PV^+^ in L2/3: Control, 2.9 ± 0.3; mDEO, 2.8 ± 0.3; unpaired *t* test, *p* = 0.7; in L4: Control, 5.2 ± 0.4; mDEO, 5.2 ± 0.4; unpaired *t* test, *p* = 0.9; in L5: Control, 4.3 ± 0.4; mDEO, 4.2 ± 0.6; unpaired *t* test, *p* = 0.8; in L6: Control, 2.1 ± 0.1; mDEO, 2.1 ± 0.2; unpaired *t* test, *p* = 0.8. Percentage of SST^+^ in L2/3: Control, 2.6 ± 0.3; mDEO, 2.7 ± 0.4; unpaired *t* test, *p* = 0.8; in L4: Control, 2.8 ± 0.2; mDEO, 3.1 ± 0.23; unpaired *t* test, *p* = 0.9; in L5: Control, 3.5 ± 0.4; mDEO, 3.8 ± 0.6; unpaired *t* test, *p* = 0.9; in L6: Control, 3.4 ± 0.2; mDEO, 3.5 ± 0.2; unpaired *t* test, *p* = 0.9).

**Figure 9.**
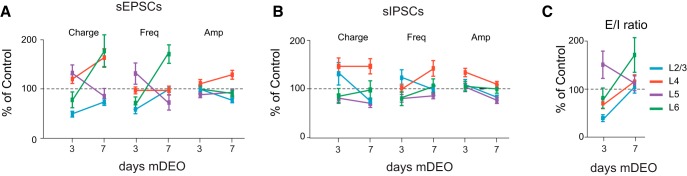
Effect of 3- and 7-d mDEO on synaptic transmission and E/I ratio. ***A***, Effect of mDEO on sEPSC charge, frequency, and amplitude plotted by layer and as percentage of control (represented by the dotted line). L2/3: blue; L4: red; L5: purple; L6: green. ***B***, Laminar changes in sIPSC charge, frequency, and amplitude induced by 3- or 7-d mDEO. ***C***, Laminar ratio of excitatory and inhibitory charge after 3- or 7-d mDEO. The statistical analyses for these data are reported in Tables 6 and 7.

Analysis of synaptic transmission, on the other hand, unveiled laminar-specific differences in the effects of mDEO. In L2/3, 3-d mDEO reduced excitatory charge through a decrease in sEPSC frequency, but no change in sEPSC average amplitude (Fig. 9*A*; Table 6). No changes in sIPSC charge, frequency, and amplitude were observed in this layer (Fig. 9*B*; Table 6). These changes resulted in a net shift of the E/I balance toward inhibition (E/I ratio in L2/3: Control, 0.60 ± 0.07, *n* = 10; mDEO P17, 0.24 ± 0.04, *n* = 11; unpaired *t* test, *p* < 0.0003). In contrast, in L4, 3-d mDEO induced no change in excitatory transmission but a significant increase in sIPSC charge and average amplitude (Fig. 9*A*, *B*; Table 6). Thus, whereas the E/I balance in L4 was shifted toward inhibition similarly to L2/3 (E/I ratio in L4: Control, 0.35 ± 0.03, *n* = 11; mDEO P17, 0.25 ± 0.03, *n* = 12; unpaired t-test *p* = 0.04), distinct synaptic mechanisms underlie the shift in E/I balance in these two superficial layers. Excitatory and inhibitory synaptic transmission onto pyramidal neurons located in L5 and L6 were not affected by 3-d mDEO (Fig. 9*A*, *B*), as demonstrated by no change to the E/I ratio after the manipulation (L5: Control, 0.24 ± 0.04, *n* = 9; mDEO P17, 0.36 ± 0.07, *n* = 12; unpaired t-test *p* = 0.2; L6: Control, 0.6 ± 0.1, *n* = 11; mDEO P17, 0.5 ± 0.1, *n* = 10; unpaired t-test *p* = 0.5). These data indicate that synaptic drive onto pyramidal neurons in the deep layers of V1 is not sensitive to brief, unilateral delays in the onset of vision.

**Table 6.** Changes in excitatory and inhibitory synaptic transmission after mDEO expressed as % change from control neurons of the hemisphere contralateral to the open eye.

Layer	sEPSC charge		sEPSC frequency		sEPSC amplitude		sIPSC charge		sIPSC frequency		sIPSC amplitude		E/I ratio	
	mDEO P17	mDEO P21	mDEO P17	mDEO P21	mDEO P17	mDEO P21	mDEO P17	mDEO P21	mDEO P17	mDEO P21	mDEO P17	mDEO P21	mDEO P17	mDEO P21
L2/3	50 ± 6*;* *p < 0.001	73 ± 6; *p = 0.03	58 ± 9; *p* = 0.003	99 ± 6; *p* = 0.9	99 ± 10; *p* = 0.9	76 ± 5; *p = 0.01	131 ± 23; *p* = 0.2	75 ± 7; *p = 0.02	123 ± 16; *p* = 0.2	98 ± 4; *p* = 0.9	108 ± 12; *p* = 0.6	83 ± 5;*p = 0.04	40 ± 7; *p < 0.001	104 ± 13; *p* = 0.7
L4	120 ± 9; *p* = 0.2	164 ± 18; *p = 0.01	97 ± 8; *p* = 0.8	97 ± 9; *p* = 0.9	111 ± 7; *p* = 0.2	130 ± 9; *p = 0.01	147 ± 16; *p = 0.01	147 ± 16; *p = 0.01	101 ± 8; *p* = 0.9	142 ± 16; *p = 0.04	134 ± 8; *p = 0.003	109 ± 7; *p* = 0.3	72 ± 9; *p = 0.04	116 ± 14; *p* = 0.4
L5	132 ± 16; *p* = 0.15	85 ± 16; *p* = 0.5	131 ± 21; *p* = 0.3	72 ± 15; *p* = 0.5	88 ± 5; *p* = 0.1	93 ± 7; *p* = 0.5	80 ± 8; *p* = 0.2	70 ± 9; *p* = 0.09	80 ± 8; *p* = 0.3	87 ± 7; *p* = 0.2	103 ± 8; *p* = 0.7	76 ± 4; *p = 0.04	160 ± 29; *p* = 0.1	112 ± 15; *p* = 0.6
L6	78 ± 16; *p* = 0.4	177 ± 33; *p = 0.02	71 ± 13; *p* = 0.2	170 ± 19 *p = 0.003	99 ± 7; *p* = 0.9	90 ± 5; *p* = 0.2	86 ± 18; *p* = 0.16	97 ± 19; *p* = 0.9	80 ± 14; *p* = 0.5	107 ± 14; *p* = 0.7	106 ± 10; *p* = 0.6	100 ± 8; *p* = 0.9	81 ± 21; *p* = 0.5	171 ± 36; *p* = 0.06

Results are expressed as mean % change ± SEM. *Statistically significant (*p* < 0.05) by *t* test.

Interestingly, if eye opening was delayed unilaterally for 7 d (from P14 to P21), the decrease in excitatory charge in L2/3 persisted, although in this case it was not due to a decrease in frequency but rather to a decrease in average sEPSC amplitude (Fig. 9*A*; Table 6). Furthermore, inhibitory charge onto L2/3 pyramidal neurons was also decreased, with a significant reduction in sIPSC amplitude (Fig. 9*B*; Table 6). The decrease in inhibition is consistent with a scaling-down effect (Kilman et al., 2002) and favors an adjustment of the E/I balance: after 7-d mDEO, the ratio of excitatory and inhibitory charge onto L2/3 pyramidal neurons returned to control levels (E/I ratio in L2/3: Control P21, 0.26 ± 0.02 (*n* = 14); mDEO P21, 0.27 ± 0.03 (*n* = 13); unpaired *t* test, *p* = 0.7).

In L4, the increase in inhibitory charge persisted if mDEO was maintained for 7 d, although the underlying mechanisms changed: at P21 there was a significant increase in sIPSC frequency, but no change in average sIPSC amplitude (Fig. 9*B*; Table 6). In this layer, prolonged mDEO also led to a significant increase in excitatory charge accompanied by an increase in average sEPSC amplitude (Fig. 9*A*; Table 6) that appear to be consistent with a scaling-up mechanism (Turrigiano et al., 1998). The homeostatic nature of the increase in excitation is supported by the fact that the E/I ratio of excitatory and inhibitory charge onto L4 pyramidal neurons returned to control levels in rats with 7-d mDEO (E/I ratio in L4: Control P21, 0.34 ± 0.05 (*n* = 12); mDEO P21, 0.39 ± 0.04 (*n* = 11); unpaired *t* test, *p* = 0.4).

In L6, there was a significant increase in sEPSC charge and frequency, but no change in sEPSC amplitude after 7-d mDEO (Fig. 9*A*; Table 6), while inhibitory drive onto L6 neurons was not significantly affected. In this layer, the E/I ratio showed a trend toward increased excitation that did not reach statistical significance (E/I ratio in L6: Control P21, 0.20 ± 0.04 (*n* = 14); mDEO P21, 0.40 ± 0.08 (*n* = 11); unpaired *t* test, *p* = 0.06). Excitatory and inhibitory synaptic drive onto L5 pyramidal neurons were not affected by mDEO of any duration, suggesting that synaptic transmission onto neurons in the output layer of V1 is not sensitive to unilateral manipulations of the time of eye opening (E/I ratio in L5: Control P21, 0.14 ± 0.02 (*n* = 10); mDEO P21, 0.15 ± 0.02 (*n* = 10); unpaired *t* test, *p* = 0.6). The percentage change in E/I ratio for mDEO P17 and mDEO P21 are reported in Fig. 9*C* (see statistical analysis in Table 6).

The layer-specific changes in E/I ratio induced by 3-d mDEO are represented in Fig. 9*C* (see also statistical analysis in Table 7); the decrease in E/I ratio was observed exclusively for L2/3 and L4 pyramidal neurons.

**Table 7.** Laminar specific effects in excitatory and inhibitory synaptic transmission following monocular delayed eye opening

mDEO	sEPSC charge	sEPSC frequency	sEPSC amplitude	sIPSC charge	sIPSC frequency	sIPSC amplitude	E/I ratio
P17							
KW-ANOVA	*p < 0.001	*p = 0.004	*p* = 0.1	*p = 0.009	*p = 0.04	*p* = 0.08	*p* < 0.001
M-W *U*							
L2/3 vs. L4	*p < 0.001	*p = 0.004		*p* = 0.5	*p* = 0.2		*p < 0.001
L2/3 vs. L5	*p < 0.001	*p = 0.003		*p* = 0.1	*p* = 0.06		*p < 0.001
L2/3 vs. L6	*p* = 0.2	*p* = 0.3		*p* = 0.2	*p* = 0.02		*p* = 0.08
L4 vs. L5	*p* = 0.6	*p* = 0.4		*p = 0.001	*p* = 0.1		*p = 0.001
L4 vs. L6	*p* = 0.01	*p* = 0.09		*p* = 0.009	*p* = 0.04		*p* = 0.01
L5 vs. L6	*p* = 0.02	*p* = 0.05		*p* = 0.9	*p* = 0.7		*p* = 0.02
P21							
KW-ANOVA	*p < 0.001	*p = 0.001	*p < 0.001	*p = 0.002	*p = 0.04	*p = 0.01	*p* = 0.5
M-W *U*							
L2/3 vs. L4	*p < 0.001	*p* = 0.7	*p < 0.001	*p < 0.001	*p* = 0.03	*p = 0.007	
L2/3 vs. L5	*p* = 0.7	*p* = 0.08	*p* = 0.1	*p* = 0.7	*p* = 0.4	*p* = 0.8	
L2/3 vs. L6	*p = 0.001	*p = 0.004	*p* = 0.1	*p* = 0.7	*p* = 0.9	*p* = 0.1	
L4 vs. L5	*p = 0.003	*p* = 0.09	*p* = 0.008	*p < 0.001	*p = 0.003	*p = 0.003	
L4 vs. L6	*p* = 0.8	*p = 0.006	*p = 0.002	*p* = 0.02	*p* = 0.1	*p* = 0.3	
L5 vs. L6	*p = 0.003	*p = 0.002	*p* = 0.7	*p* = 0.5	*p* = 0.5	*p* = 0.09	

*Statistically significant. Only *p* values <0.008 are considered significant due to Bonferroni correction for multiple comparisons. Values for mDEO P17 and mDEOP21 are reported in Table 6.

All the effects reported above were not due to changes in input resistance, as this parameter was not affected by mDEO in any recorded neuron at either P17 or P21. R_in_ (MΩ) in L2/3: P17 Control, 79 ± 5, *n* = 10; 3-d mDEO, 71 ± 2 (*n* = 11); unpaired *t* test, *p* = 0.6; P21 Control, 79 ± 5 (*n* = 14); 7-d mDEO, 80 ± 5 (*n* = 13); *p* = 0.9; in L4: P17 Control, 95 ± 6 (*n* = 11); 3-d mDEO, 106 ± 4 (*n* = 12); *p* = 0.2; P21 Control, 102 ± 12 (*n* = 11); 7-d mDEO, 87 ± 5 (*n* = 12); *p* = 0.3; in L5: P17 Control, 67 ± 6 (*n* = 9); 3-d mDEO, 70 ± 3 (*n* = 12); *p* = 0.7; P21 Control, 70 ± 10 (*n* = 10); 7-d mDEO, 72 ± 9 (*n* = 10); *p* = 0.9; in L6: P17 Control, 121 ± 9 (*n* = 11); 3-d mDEO, 136 ± 10 (*n* = 10); *p* = 0.25; P21 Control, 113 ± 7 (*n* = 14); 7-d mDEO, 115 ± 9 (*n* = 11); *p* = 0.8.

Our data indicate that mDEO has profound effects on V1 that not only are laminar specific, but also depend on the duration of the manipulation. Although the effect of 3-d mDEO was to shift the E/I balance toward inhibition in both L2/3 and L4, a longer manipulation restored the E/I ratio to control levels in both layers. The readjustment of the E/I balance was not dependent on the restoration of synaptic transmission to control levels, but on the recruitment of additional synaptic changes.

Taken together, our results demonstrate that bDEO and mDEO have profoundly different effects on V1 circuitry. Whereas bDEO alters synaptic transmission while maintaining E/I balance and coordination across layers, mDEO disrupts the coordination of superficial and deep layers and significantly affects the excitability of local circuits by shifting the E/I ratio. Altogether, our results suggest that shifts in the time of eye opening trigger multistep cascades of events that involve laminar-specific mechanisms for synaptic plasticity.

## Discussion

We have shown that at the time of eye opening, excitatory and inhibitory circuits in V1 begin a laminar-specific maturation process that include changes in the percentage of PV^+^ neurons, as well as plasticity of synaptic transmission onto pyramidal neurons. Before eye opening, excitatory and inhibitory drive onto pyramidal neurons in primary thalamorecipient layers (L4 and L6) are excitation shifted or balanced. From the time of eye opening to the peak of the critical period, the E/I ratio shifts toward similar levels of dominant inhibition through the maturation of synaptic transmission (Heinen et al., 2004; Chattopadhyaya et al., 2007) and, in L2/3, L4, and L6, a progressive increase in the percentage of PV^+^ neurons (Gonchar et al., 2007). In contrast, in L5, the number of PV^+^ and SST^+^ neurons is stable during the P14–P30 window, suggesting that the shift toward inhibition in this layer is primarily due to maturation of synaptic transmission (Chattopadhyaya et al., 2007; Huang, 2009). Interestingly, in L2/3, the number of PV^+^ neurons before eye opening is low, but the E/I balance is already dominated by inhibition, suggesting that early in postnatal development SST^+^ neurons may be the dominant source of inhibition in this layer. Alternatively, L2/3 pyramidal neurons may receive inhibitory inputs from other groups of GABAergic neurons (Taniguchi et al., 2013). The increase in number of PV^+^ neurons during postnatal development was previously reported in mouse V1 (Gonchar et al., 2007). However, Gonchar et al. (2007) reported few PV^+^ neurons in L5 at eye opening and a progressive increase in PV^+^, as well as SST^+^ neurons, from the time of eye opening to the peak of the critical period. In rat V1, we report that the number of PV^+^ neurons in L5 is stable at eye opening, and the number of SST^+^ neurons does not change in any layer in the developmental window under study. This discrepancy could be due to species-specific differences or in the subregion of V1 in which the analysis was performed: we restricted our analysis to the monocular portion of V1 (V1m), whereas the previous study did not specify the location under analysis. The increase in number of PV^+^ neurons in V1 is likely dependent on a developmental regulation in the expression of PV, as fate-mapping studies report that the migration of neurons from the medial ganglionic eminence, the primary source of inhibitory neurons to cortex, is complete by eye opening (Wichterle et al., 2001).

Analyses of the amplitude and frequency of spontaneous synaptic currents were instrumental in identifying laminar differences in the maturation of excitatory and inhibitory drive. Indeed, even in the absence of developmental changes in excitatory charge, there were underlying maturation processes expressed presynaptically, postsynaptically, or both. These data are consistent with recent findings regarding the contribution of glutamatergic synapse maturation to critical period plasticity (Rumpel et al., 1998, 2004; Huang et al., 2015). Conversely, the maturation of inhibitory drive appears to follow similar trends in all layers: inhibitory charge increased primarily owing to a higher frequency of sIPSC and no changes in the average sIPSC amplitude. This effect suggests a presynaptic site for the maturation of inhibitory drive, consistent with previous findings (Chattopadhyaya et al., 2007; Wu et al., 2012). The shift toward an increased proportion of large sIPSC in the cumulative distribution, as well as the absence of changes in the average amplitude, agree with an increase in release probability. The input resistance of pyramidal neurons in all layers decreased over the developmental window under study (except for L5, where it remained stable), indicating that the increase in inhibitory drive cannot be explained by a change in membrane properties, but rather depends on maturation of synaptic transmission. Taken together, these data support the interpretation that pyramidal neurons in V1 engage layer-specific mechanisms of maturation during the third and fourth week after eye opening. Interestingly, the patterns of maturation in primary thalamorecipient layers (L4 and L6) differ from those for L2/3 and L5, suggesting that the organization of long-range inputs onto V1 neurons may play a role in the maturation process.

### Time of eye opening and maturation of synaptic transmission

Manipulations of the time of eye opening allowed us to distinguish developmentally regulated events that depend on the onset of visual experience from others that are independent of the onset of vision. Binocular delayed eye opening (bDEO) and monocular delayed eye opening (mDEO) did not affect the percentage of PV^+^ neurons in V1, indicating that this parameter is independent of visual experience. This effect is in apparent contrast with previous findings. The discrepancies, however, are likely due to several factors. We used delayed eye opening by eyelid suture instead of deafferentation (Schmidt-Kastner et al., 1992), dark rearing, or monocular deprivation after eye opening (Tropea et al., 2006). In addition, we counted the number of PV^+^ somata, whereas previous studies analyzed the level of PV immunoreactivity across V1 (Cellerino et al., 1992; Schmidt-Kastner et al., 1992) or the cellular level of expression of parvalbumin (Tropea et al., 2006). Finally, our analysis was restricted to the monocular portion of V1, whereas previous studies included monocular and binocular regions or did not specify the area selected for analysis. This is a relevant issue, as studies have shown that visual deprivation can drive comparatively different changes in PV expression in binocular and monocular V1 (Cellerino et al., 1992). In our preparation, the number of SST^+^ neurons was not affected by either development or manipulations of the time of eye opening, indicating that SST^+^ neuron-mediated inhibition is a reliable source of inhibition in all layers of V1 throughout development.

Synaptic transmission onto pyramidal neurons was strongly modulated by the time of eye opening. Binocular or monocular eye opening delays of different durations were instrumental for revealing the capacity of V1 to engage distinct, laminar-specific mechanisms for experience-dependent rewiring. A 3-d binocular delayed eye opening reduced excitatory and inhibitory transmission with similar trends across V1 layers, apart from L6. The effects of this manipulation were fully compensated for when eye opening was delayed by 7 d. These results are consistent with previous work showing that delays in the onset of vision can delay physiologic plasticity in V1 (Mower et al., 1985), and with previous work showing that neurons in V1 can engage compensatory, or homeostatic, mechanisms in response to changes in visual drive (Desai et al., 2002; Maffei et al., 2004; Maffei and Turrigiano, 2008; Lambo and Turrigiano, 2013). Despite the changes in synaptic drive, 3- and 7-d bDEO did not shift the E/I balance of synaptic charge onto pyramidal neurons in any of the layers, suggesting that if eye opening is delayed bilaterally, the circuit in V1 has the capacity to readjust its state of excitability and preserve coordination of synaptic transmission across layers. This interpretation is consistent with previous work showing that V1 neurons have the capacity to recover control-level firing rates if visual manipulations are maintained for several days (Hengen et al., 2013; Keck et al., 2013; Lambo and Turrigiano, 2013).

On the other hand, unilateral delays in eye opening had laminar-specific effects. The impact of 3-d mDEO was significant not only on synaptic transmission, but also on the E/I ratio of charges onto pyramidal neurons in the superficial layers of V1, and on the coordination of synaptic changes across layers. Although increasing the duration of mDEO to 7 d recovered the E/I balance onto pyramidal neurons in all layers, changes in synaptic transmission persisted. These results suggest that binocular visual drive at the time of eye opening is necessary to preserve coordination across layers. After an initial shift in E/I balance in the superficial layers, additional plastic changes were triggered to compensate for the shift in E/I ratio, although overall synaptic transmission did not return to control. In L2/3, the decrease in excitatory drive induced by 3-d mDEO was compensated for by a decrease in inhibitory drive observed with 7-d mDEO, consistent with the recruitment of a scaling-down mechanism for inhibition (Kilman et al., 2002). In contrast, in L4, the initial increase in inhibition was compensated by an increase in the average amplitude of sEPSC, consistent with a scaling-up mechanism (Turrigiano et al., 1998). The circuit mechanisms recruited by a long-lasting mDEO can be considered compensatory, as their engagement restored the E/I ratio to control (Maffei and Fontanini, 2009). Despite the recovery of the E/I ratio, synaptic transmission in V1 remained altered after 7-d mDEO, suggesting that homeostatic plasticity stabilized excitability but did not recover control levels of synaptic transmission as we observed for bDEO. Restoration of E/I balance without recovery of synaptic drive has been proposed as a mechanism for circuit dysfunction underlying pathologic conditions (Lewis et al., 2012) and could potentially explain long-term effects of early unilateral visual deprivation on visual function (van Sluyters, 1978a, b).

Taken together, our data indicate that the effects of mDEO onto V1 pyramidal neurons are laminar specific and long-lasting, likely engaging mechanisms distinct from those recruited by the short-lasting effects of bDEO. Our results demonstrate that coordination of developmental patterns of maturation can be disrupted, especially if the visual input to the eyes is unbalanced.

### Implications of laminar-specific refinement for the development of visual function

Delayed eye opening early in life is known to have dramatic effects on the organization of the visual system. Children with congenital binocular or monocular cataracts show visual impairments that can have lasting consequences even after corrective surgery (Lewis and Maurer, 2009). Studies in primates point to impaired development of V1 neuron response properties as the leading cause of long-lasting visual impairment (Crawford et al., 1975; Blakemore and Vitaldurand, 1983). Indeed, the receptive field properties of V1 neurons are altered by both binocular (Ellemberg et al., 1999) and monocular (Ellemberg et al., 2000) delayed eye opening.

The consequences of delayed eye opening extend beyond altered receptive field properties, however, affecting sensitivity to high spatial frequencies and sensitivity to feature spacing that alter the perception of motion (Ellemberg et al., 2000). Monocular delayed eye opening has more dramatic effects than binocular delayed eye opening on the response properties of V1 neurons; however, the opposite has been reported for motion perception (Ellemberg et al., 2002).

The different patterns of experience-dependent reorganization of laminar circuits we report for mDEO and bDEO suggest that distinct synaptic and circuit mechanisms may be involved in the development of different aspects of healthy visual function. One may speculate that the ability of V1 neurons to recruit compensatory mechanisms to recover activity after long-lasting binocular deprivation may facilitate recovery of V1 neuron response properties if the eyes are reopened. On the contrary, the reorganization of local circuits after monocular delayed eye opening may induce long-lasting changes in V1 neurons response properties, as synaptic changes persist even after the E/I balance has recovered to control levels. The results reported in our study support the idea that healthy visual function relies on the coordinated development of synaptic activity in all layers of V1. Disruption of such coordination may be the mechanism leading to persistent impairment in visual function even after incoming visual drive is restored.

## Synthesis

Reviewing Editor: Christian Hansel, University of Chicago Decisions are customarily a result of the Reviewing Editor and the peer reviewers coming together and discussing their recommendations until a consensus is reached. When revisions are invited, a fact-based synthesis statement explaining their decision and outlining what is needed to prepare a revision will be listed below. Note: If this manuscript was transferred from JNeurosci and a decision was made to accept the manuscript without peer review, a brief statement to this effect will instead be what is listed below.

The previous critiques of two reviewers selected by the Journal of Neuroscience have been appropriately addressed.
